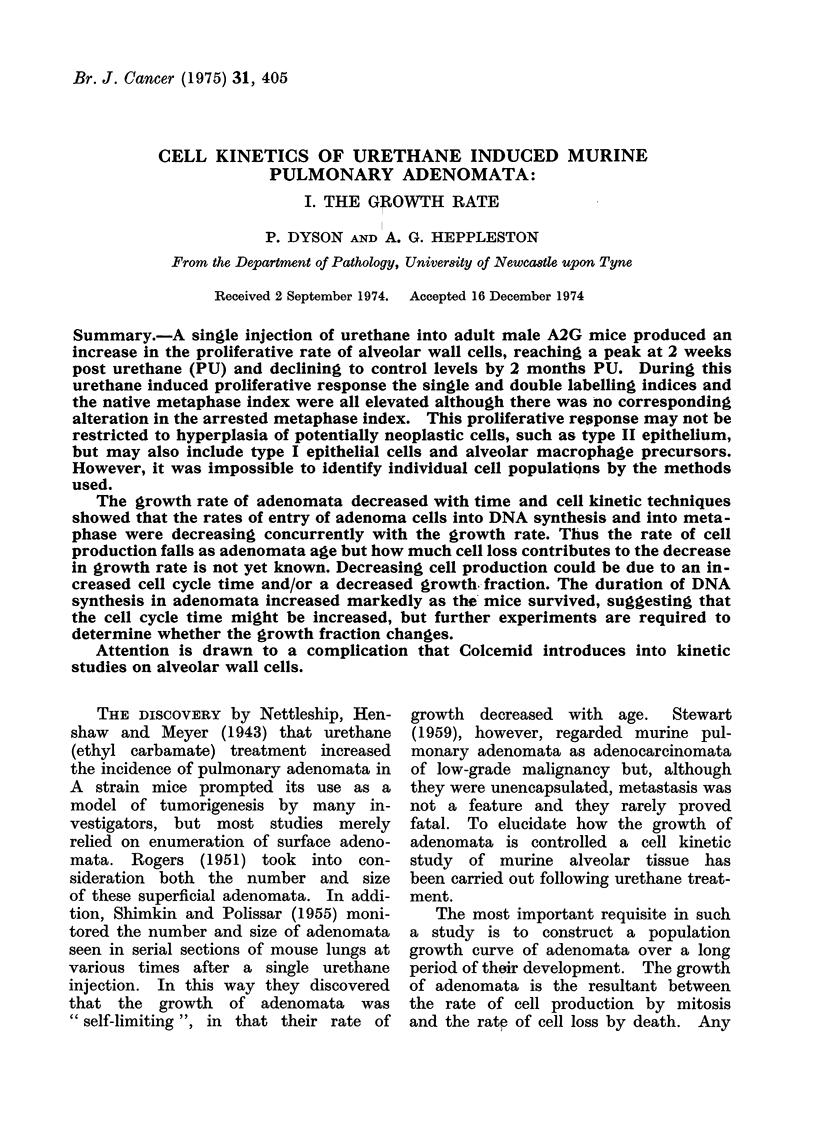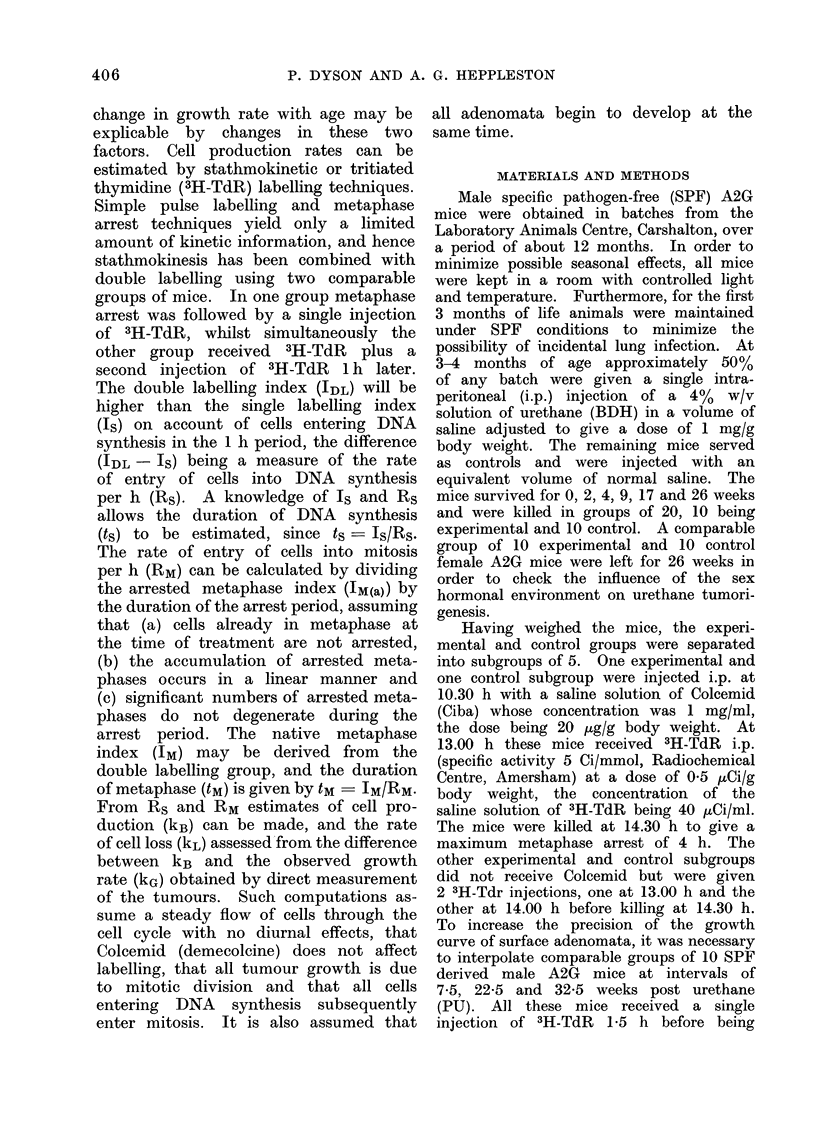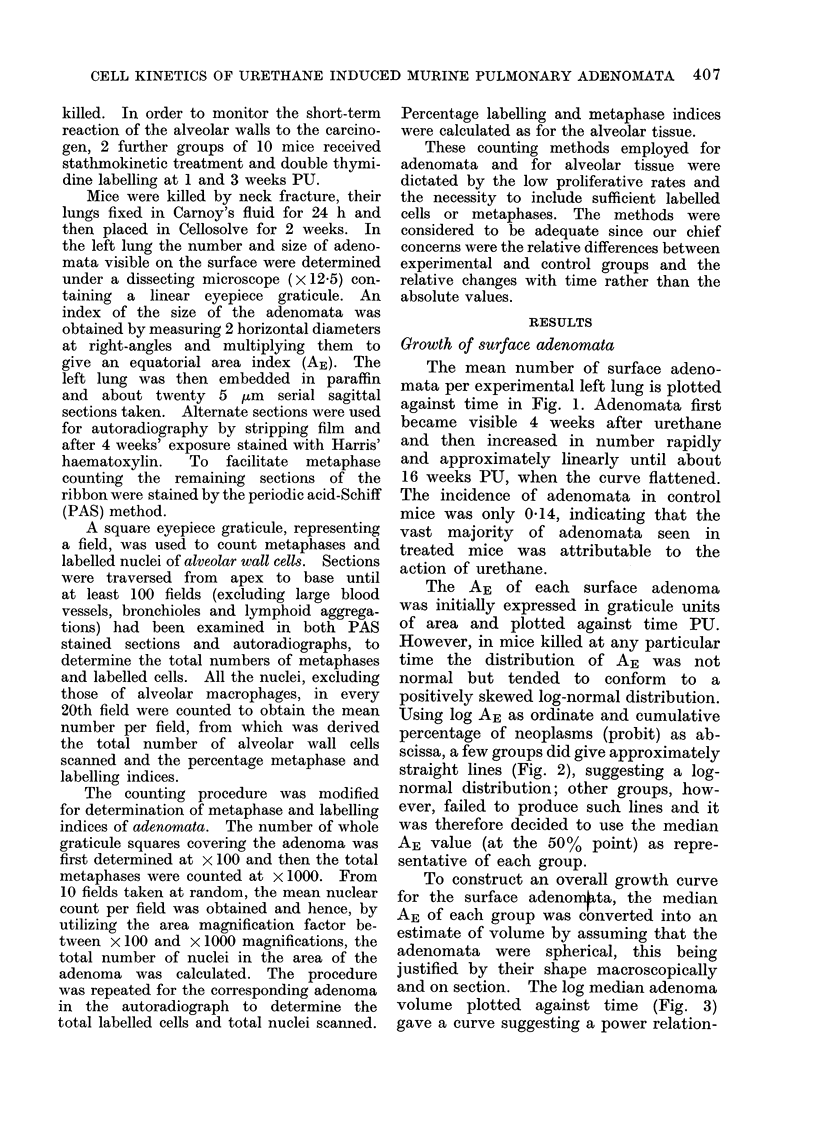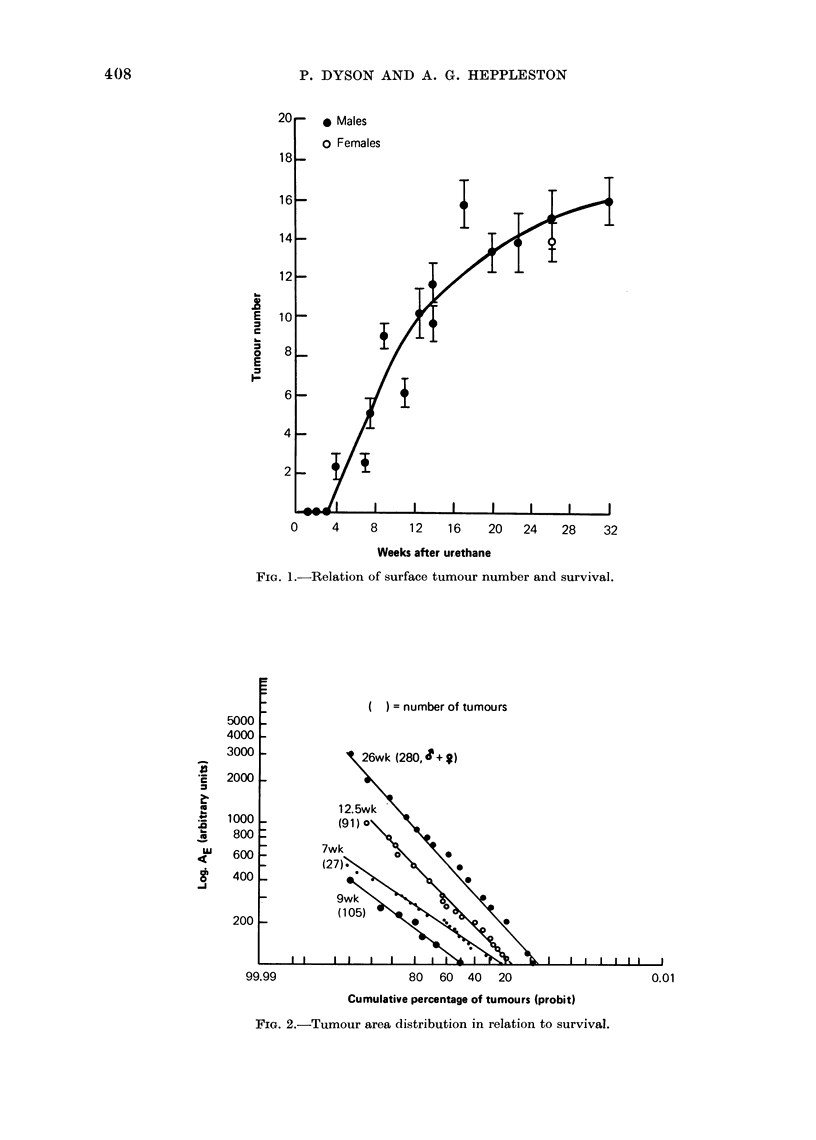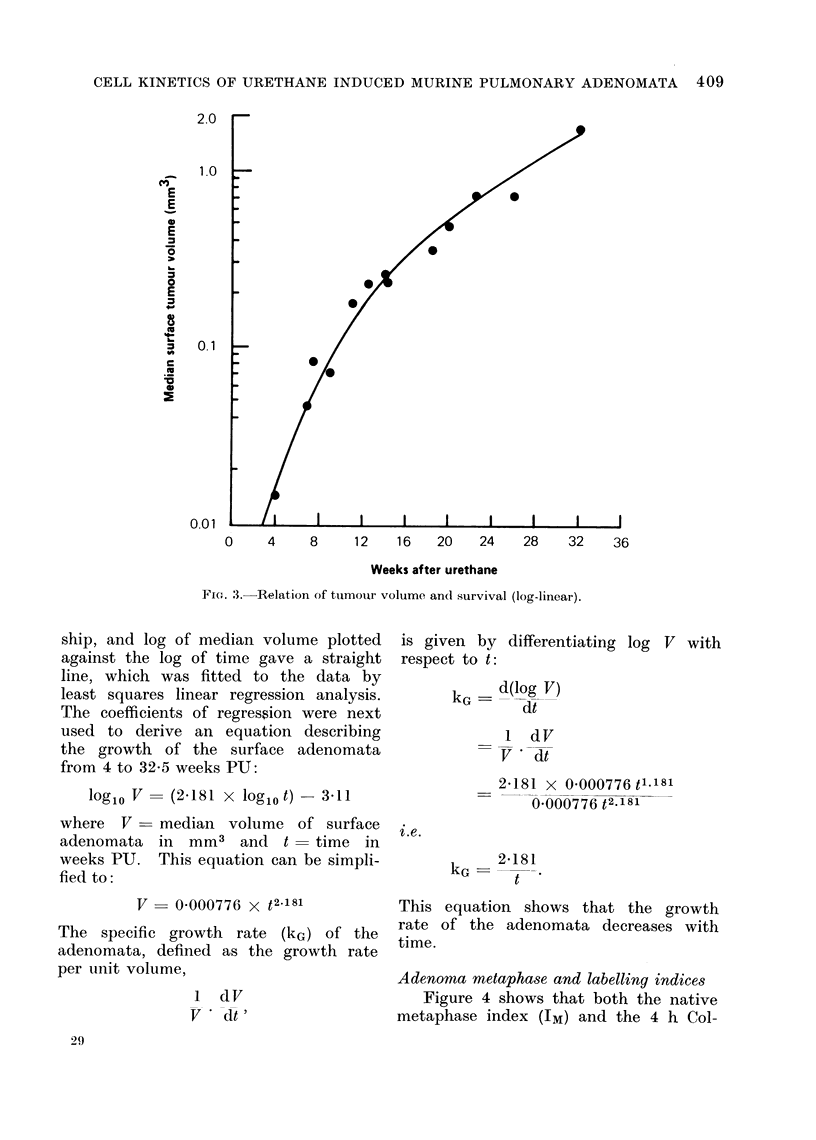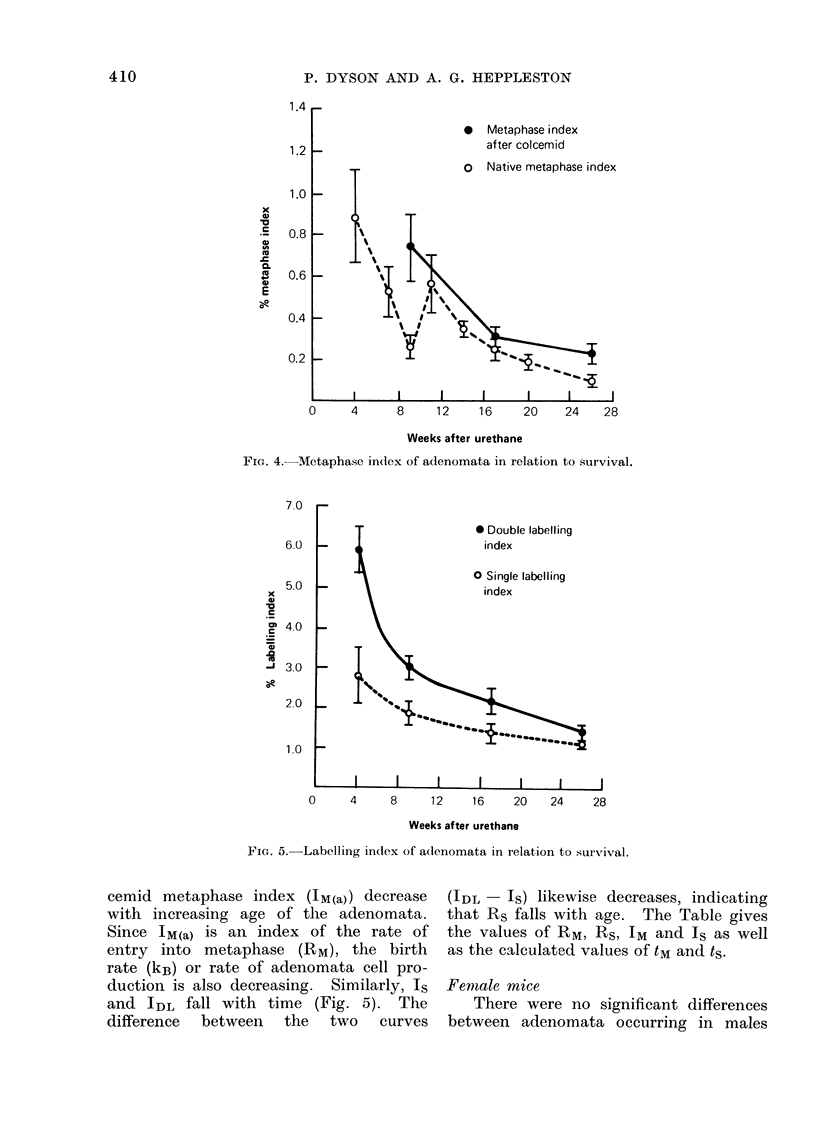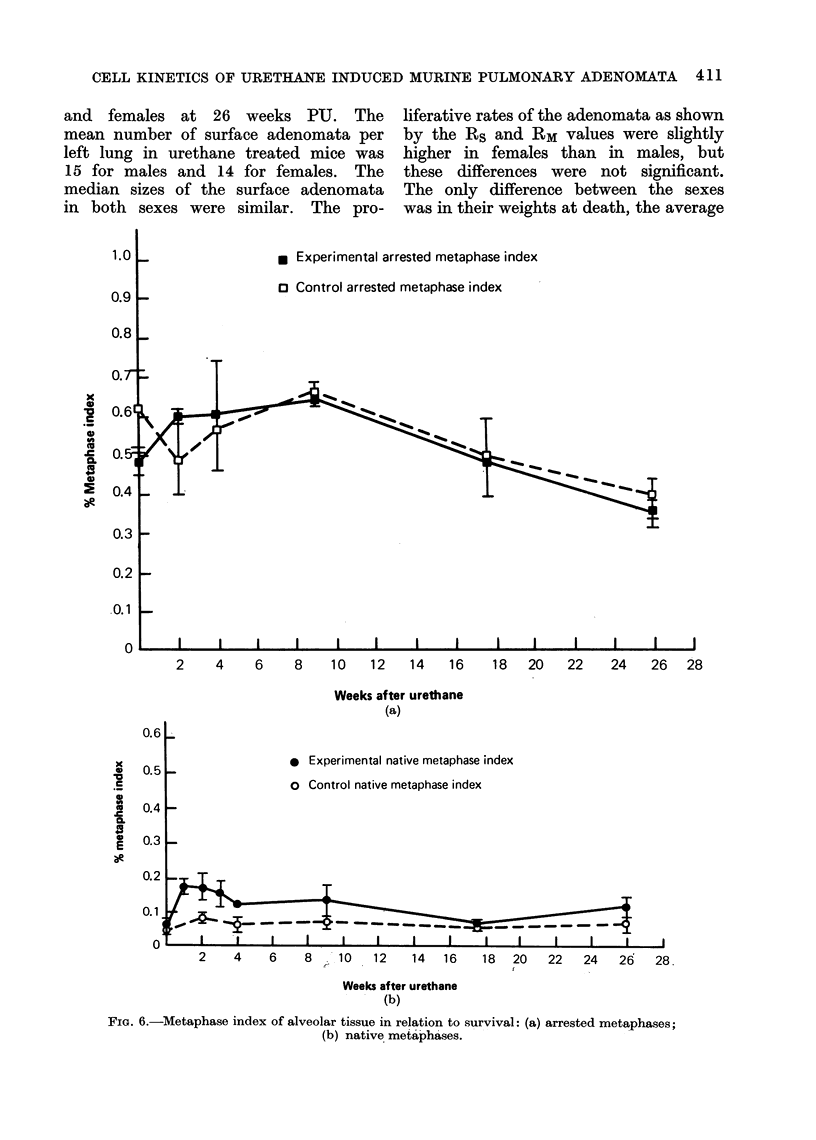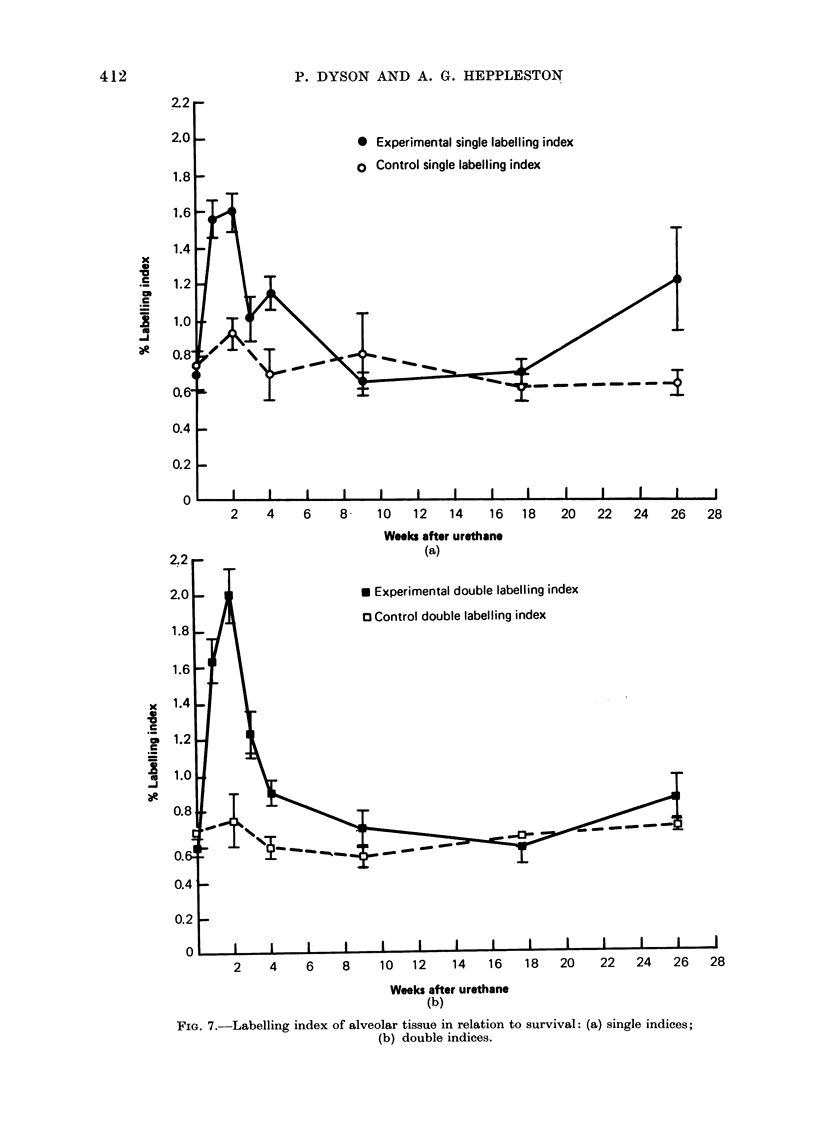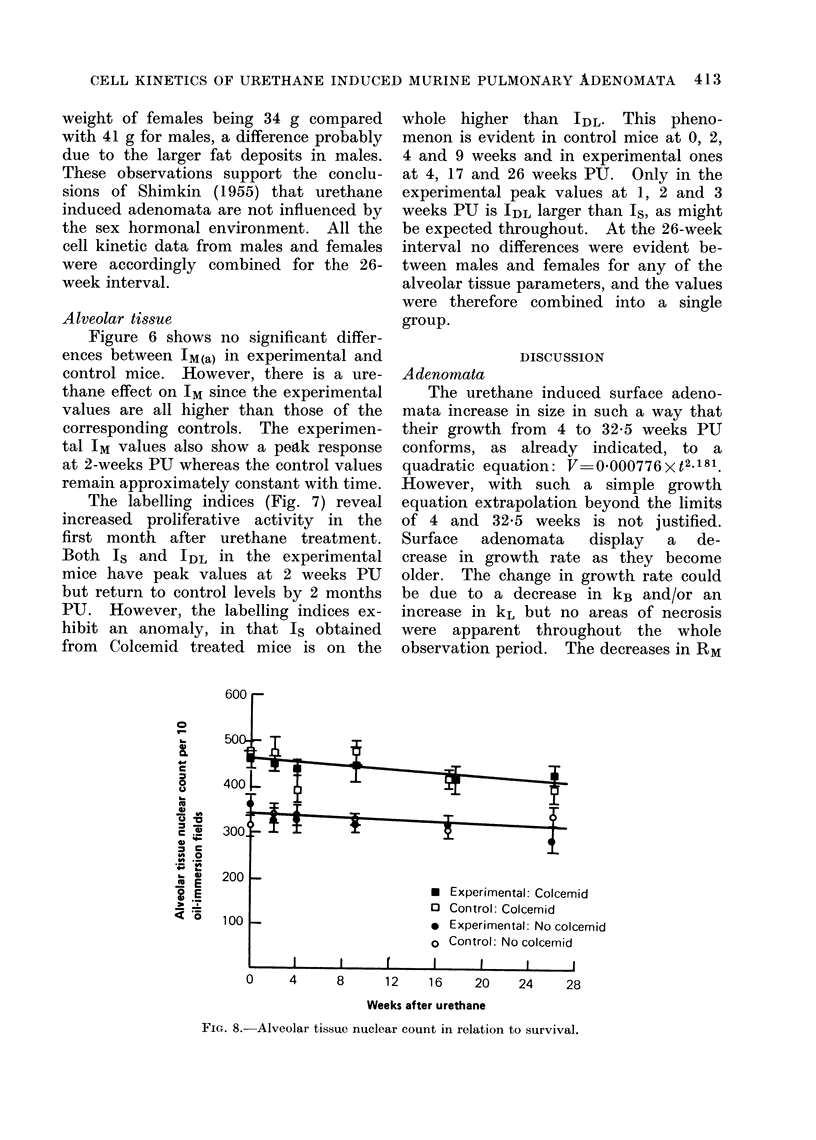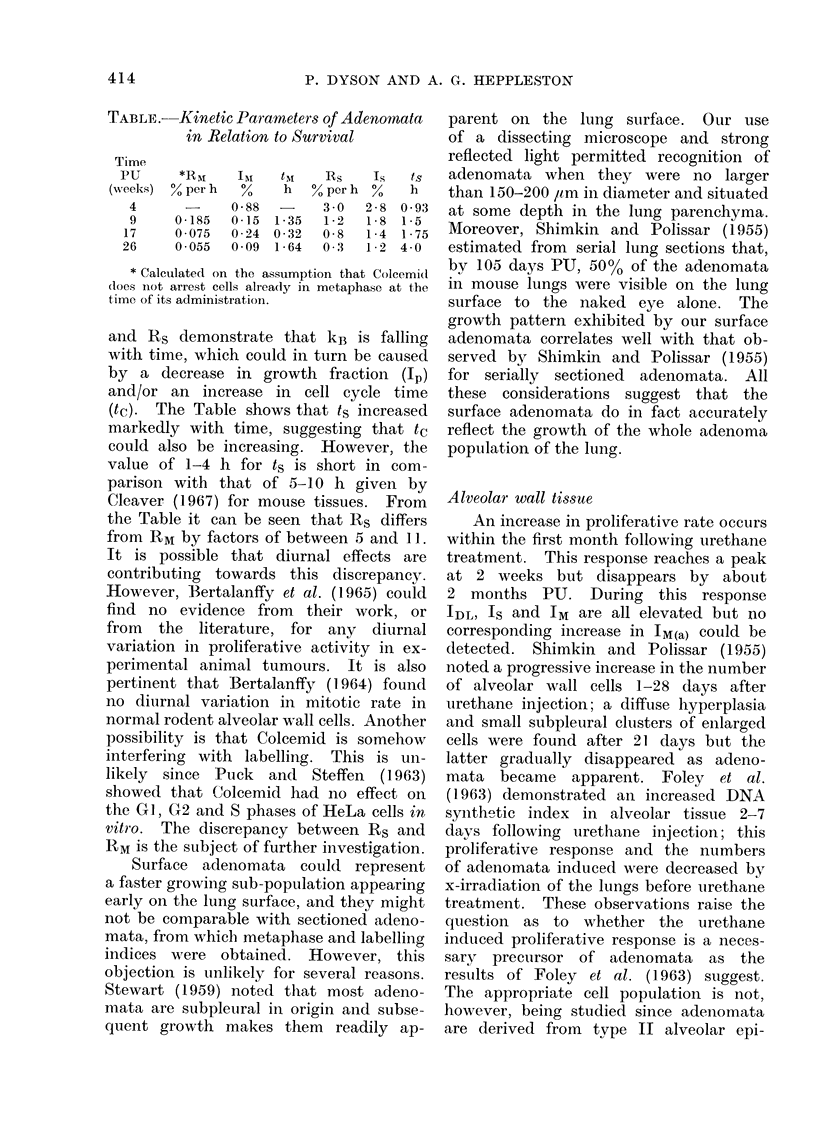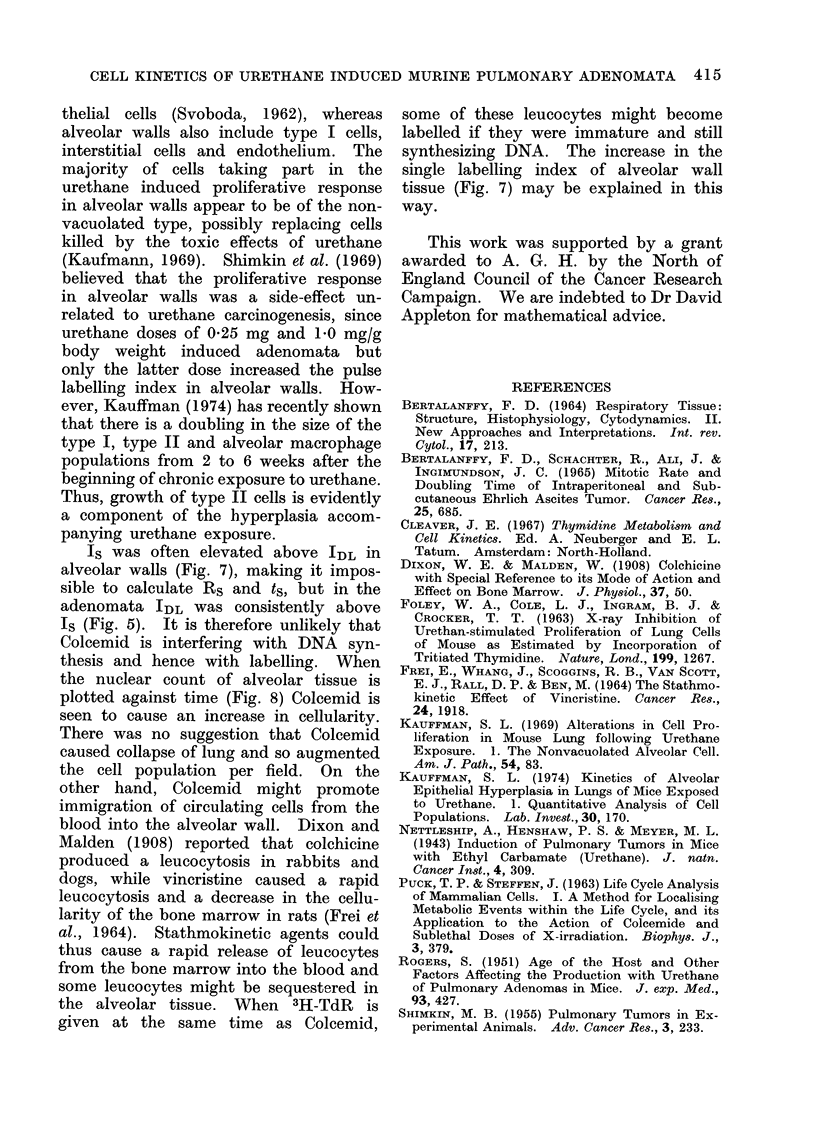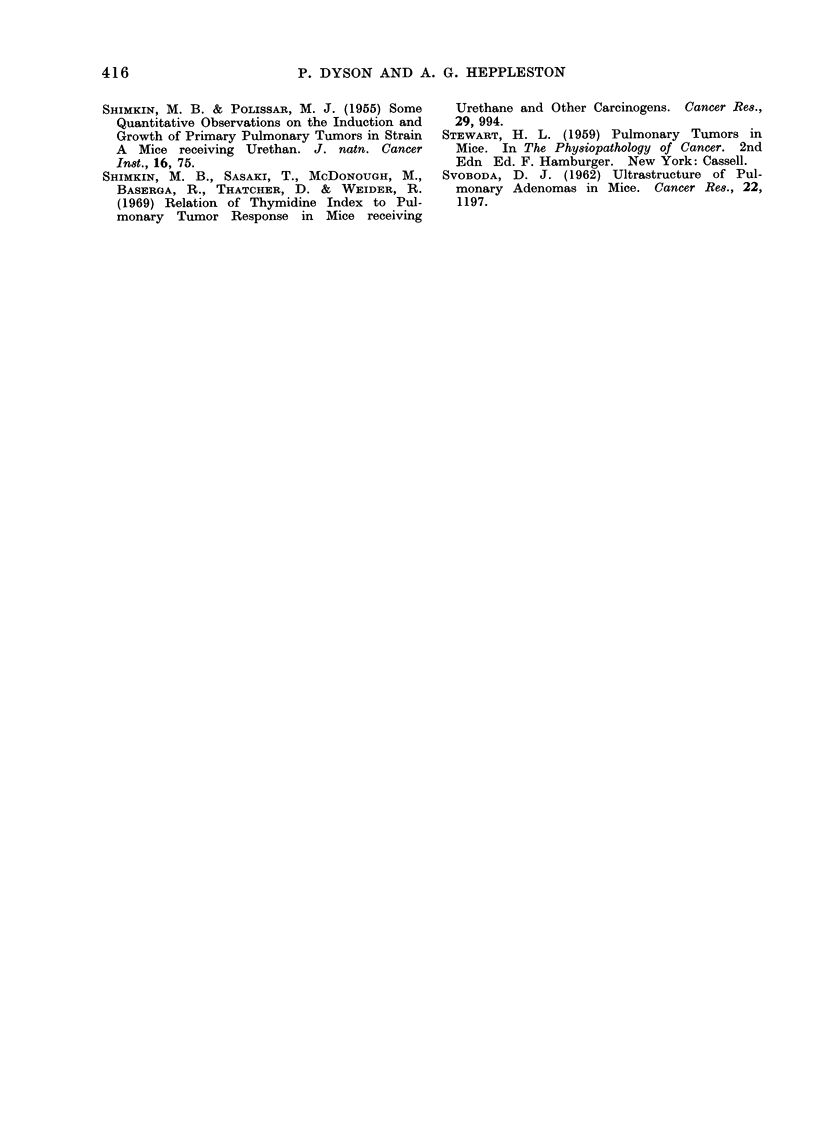# Cell kinetics of urethane induced murine pulmonary adenomata: I. The growth rate.

**DOI:** 10.1038/bjc.1975.79

**Published:** 1975-04

**Authors:** P. Dyson, A. G. Heppleston

## Abstract

A single injection of urethane into adult male A2G mice produced an increase in the proliferative rate of alveolar wall cells, reaching a peak at 2 weeks post urethan (PU) and declining to control levels by 2 months PU. During this urethane induced proliferative response the single and double labelling indices and the native metaphase index were all elevated although there was no corresponding alteration in the arrested metaphase index. This proliferative response may not be restricted to hyperplasia of potentially neoplastic cells, such as type II epithelium, but may also include type I epithelial cells and alveolar macrophage precursors. However, it was impossible to identify individual cell populations by methods used. The growth rate of adenomata decrease with time and cell kinetic techniques showed that the rates of entry of adenoma cells into DNA synthesis and into metaphase were decreasing concurrently with the growth rate. Thus the rate of cell production falls as adenomata age but how much cell loss contributes to the decrease in growth rate is not yet known. Decreasing cell production could be due to an increased cell cycle time and/or a decreased growth fraction. The duration of DNA synthesis in adenomata increased markedly as the mice survived, suggesting that the cell cycle time might be increased, but further experiments are required to determine whether the growth fraction changes. Attention is drawn to a complication that Colcemid introduces into kinetic studies on alveolar wall cells.


*Br. J. Cancer* (1975) **31**, 405

### CELL KINETICS OF URETHANE INDUCED MURINE PULMONARY ADENOMATA:

### I. THE GROWTH RATE

P. DYSON AND A. G. HEPPLESTON

*From the Department of Pathology, University of Newcastle upon Tyne*

Received 2 September 1974. Accepted 16 December 1974

**Summary.—A single injection of urethane into adult male A2G mice produced an increase in the proliferative rate of alveolar wall cells, reaching a peak at 2 weeks post urethane (PU) and declining to control levels by 2 months PU. During this urethane induced proliferative response the single and double labelling indices and the native metaphase index were all elevated although there was no corresponding alteration in the arrested metaphase index. This proliferative response may not be restricted to hyperplasia of potentially neoplastic cells, such as type II epithelium, but may also include type I epithelial cells and alveolar macrophage precursors. However, it was impossible to identify individual cell populations by the methods used.**

**The growth rate of adenomata decreased with time and cell kinetic techniques showed that the rates of entry of adenoma cells into DNA synthesis and into metaphase were decreasing concurrently with the growth rate. Thus the rate of cell production falls as adenomata age but how much cell loss contributes to the decrease in growth rate is not yet known. Decreasing cell production could be due to an increased cell cycle time and/or a decreased growth fraction. The duration of DNA synthesis in adenomata increased markedly as the mice survived, suggesting that the cell cycle time might be increased, but further experiments are required to determine whether the growth fraction changes.**

**Attention is drawn to a complication that Colcemid introduces into kinetic studies on alveolar wall cells.**

THE DISCOVERY by Nettleship, Henshaw and Meyer (1943) that urethane (ethyl carbamate) treatment increased the incidence of pulmonary adenomata in A strain mice prompted its use as a model of tumorigenesis by many investigators, but most studies merely relied on enumeration of surface adenomata. Rogers (1951) took into consideration both the number and size of these superficial adenomata. In addition, Shimkin and Polissar (1955) monitored the number and size of adenomata seen in serial sections of mouse lungs at various times after a single urethane injection. In this way they discovered that the growth of adenomata was "self-limiting", in that their rate of growth decreased with age. Stewart (1959), however, regarded murine pulmonary adenomata as adenocarcinomata of low-grade malignancy but, although they were unencapsulated, metastasis was not a feature and they rarely proved fatal. To elucidate how the growth of adenomata is controlled a cell kinetic study of murine alveolar tissue has been carried out following urethane treatment.

The most important requisite in such a study is to construct a population growth curve of adenomata over a long period of their development. The growth of adenomata is the resultant between the rate of cell production by mitosis and the rate of cell loss by death. Any change in growth rate with age may be explicable by changes in these two factors. Cell production rates can be estimated by stathmokinetic or tritiated thymidine ($^3$H-TdR) labelling techniques. Simple pulse labelling and metaphase arrest techniques yield only a limited amount of kinetic information, and hence stathmokinesis has been combined with double labelling using two comparable groups of mice. In one group metaphase arrest was followed by a single injection of $^3$H-TdR, whilst simultaneously the other group received $^3$H-TdR plus a second injection of $^3$H-TdR 1 h later. The double labelling index ($I_{DL}$) will be higher than the single labelling index ($I_S$) on account of cells entering DNA synthesis in the 1 h period, the difference ($I_{DL} - I_S$) being a measure of the rate of entry of cells into DNA synthesis per h ($R_S$). A knowledge of $I_S$ and $R_S$ allows the duration of DNA synthesis ($t_S$) to be estimated, since $t_S = I_S/R_S$. The rate of entry of cells into mitosis per h ($R_M$) can be calculated by dividing the arrested metaphase index ($I_{M(a)}$) by the duration of the arrest period, assuming that (a) cells already in metaphase at the time of treatment are not arrested, (b) the accumulation of arrested metaphases occurs in a linear manner and (c) significant numbers of arrested metaphases do not degenerate during the arrest period. The native metaphase index ($I_M$) may be derived from the double labelling group, and the duration of metaphase ($t_M$) is given by $t_M = I_M/R_M$. From $R_S$ and $R_M$ estimates of cell production ($k_B$) can be made, and the rate of cell loss ($k_L$) assessed from the difference between $k_B$ and the observed growth rate ($k_G$) obtained by direct measurement of the tumours. Such computations assume a steady flow of cells through the cell cycle with no diurnal effects, that Colcemid (demecolcine) does not affect labelling, that all tumour growth is due to mitotic division and that all cells entering DNA synthesis subsequently enter mitosis. It is also assumed that all adenomata begin to develop at the same time.

### MATERIALS AND METHODS

Male specific pathogen-free (SPF) A2G mice were obtained in batches from the Laboratory Animals Centre, Carshalton, over a period of about 12 months. In order to minimize possible seasonal effects, all mice were kept in a room with controlled light and temperature. Furthermore, for the first 3 months of life animals were maintained under SPF conditions to minimize the possibility of incidental lung infection. At 3–4 months of age approximately 50% of any batch were given a single intraperitoneal (i.p.) injection of a 4% w/v solution of urethane (BDH) in a volume of saline adjusted to give a dose of 1 mg/g body weight. The remaining mice served as controls and were injected with an equivalent volume of normal saline. The mice survived for 0, 2, 4, 9, 17 and 26 weeks and were killed in groups of 20, 10 being experimental and 10 control. A comparable group of 10 experimental and 10 control female A2G mice were left for 26 weeks in order to check the influence of the sex hormonal environment on urethane tumorigenesis.

Having weighed the mice, the experimental and control groups were separated into subgroups of 5. One experimental and one control subgroup were injected i.p. at 10.30 h with a saline solution of Colcemid (Ciba) whose concentration was 1 mg/ml, the dose being 20 $\mu$g/g body weight. At 13.00 h these mice received $^3$H-TdR i.p. (specific activity 5 Ci/mmol, Radiochemical Centre, Amersham) at a dose of 0·5 $\mu$Ci/g body weight, the concentration of the saline solution of $^3$H-TdR being 40 $\mu$Ci/ml. The mice were killed at 14.30 h to give a maximum metaphase arrest of 4 h. The other experimental and control subgroups did not receive Colcemid but were given 2 $^3$H-Tdr injections, one at 13.00 h and the other at 14.00 h before killing at 14.30 h. To increase the precision of the growth curve of surface adenomata, it was necessary to interpolate comparable groups of 10 SPF derived male A2G mice at intervals of 7·5, 22·5 and 32·5 weeks post urethane (PU). All these mice received a single injection of $^3$H-TdR 1·5 h before being killed. In order to monitor the short-term reaction of the alveolar walls to the carcinogen, 2 further groups of 10 mice received stathmokinetic treatment and double thymidine labelling at 1 and 3 weeks PU.

Mice were killed by neck fracture, their lungs fixed in Carnoy's fluid for 24 h and then placed in Cellosolve for 2 weeks. In the left lung the number and size of adenomata visible on the surface were determined under a dissecting microscope (×12·5) containing a linear eyepiece graticule. An index of the size of the adenomata was obtained by measuring 2 horizontal diameters at right-angles and multiplying them to give an equatorial area index ($A_E$). The left lung was then embedded in paraffin and about twenty 5 $\mu$m serial sagittal sections taken. Alternate sections were used for autoradiography by stripping film and after 4 weeks' exposure stained with Harris' haematoxylin. To facilitate metaphase counting the remaining sections of the ribbon were stained by the periodic acid-Schiff (PAS) method.

A square eyepiece graticule, representing a field, was used to count metaphases and labelled nuclei of *alveolar wall cells*. Sections were traversed from apex to base until at least 100 fields (excluding large blood vessels, bronchioles and lymphoid aggregations) had been examined in both PAS stained sections and autoradiographs, to determine the total numbers of metaphases and labelled cells. All the nuclei, excluding those of alveolar macrophages, in every 20th field were counted to obtain the mean number per field, from which was derived the total number of alveolar wall cells scanned and the percentage metaphase and labelling indices.

The counting procedure was modified for determination of metaphase and labelling indices of *adenomata*. The number of whole graticule squares covering the adenoma was first determined at ×100 and then the total metaphases were counted at ×1000. From 10 fields taken at random, the mean nuclear count per field was obtained and hence, by utilizing the area magnification factor between ×100 and ×1000 magnifications, the total number of nuclei in the area of the adenoma was calculated. The procedure was repeated for the corresponding adenoma in the autoradiograph to determine the total labelled cells and total nuclei scanned. Percentage labelling and metaphase indices were calculated as for the alveolar tissue.

These counting methods employed for adenomata and for alveolar tissue were dictated by the low proliferative rates and the necessity to include sufficient labelled cells or metaphases. The methods were considered to be adequate since our chief concerns were the relative differences between experimental and control groups and the relative changes with time rather than the absolute values.

### RESULTS

### *Growth of surface adenomata*

The mean number of surface adenomata per experimental left lung is plotted against time in Fig. 1. Adenomata first became visible 4 weeks after urethane and then increased in number rapidly and approximately linearly until about 16 weeks PU, when the curve flattened. The incidence of adenomata in control mice was only 0·14, indicating that the vast majority of adenomata seen in treated mice was attributable to the action of urethane.

The $A_E$ of each surface adenoma was initially expressed in graticule units of area and plotted against time PU. However, in mice killed at any particular time the distribution of $A_E$ was not normal but tended to conform to a positively skewed log-normal distribution. Using log $A_E$ as ordinate and cumulative percentage of neoplasms (probit) as abscissa, a few groups did give approximately straight lines (Fig. 2), suggesting a log-normal distribution; other groups, however, failed to produce such lines and it was therefore decided to use the median $A_E$ value (at the 50% point) as representative of each group.

To construct an overall growth curve for the surface adenomata, the median $A_E$ of each group was converted into an estimate of volume by assuming that the adenomata were spherical, this being justified by their shape macroscopically and on section. The log median adenoma volume plotted against time (Fig. 3) gave a curve suggesting a power relation-

FIG. 1.—Relation of surface tumour number and survival.

FIG. 2.—Tumour area distribution in relation to survival.

FIG. 3.—Relation of tumour volume and survival (log-linear).

ship, and log of median volume plotted against the log of time gave a straight line, which was fitted to the data by least squares linear regression analysis. The coefficients of regression were next used to derive an equation describing the growth of the surface adenomata from 4 to 32·5 weeks PU:

$$\log_{10} V = (2{\cdot}181 \times \log_{10} t) - 3{\cdot}11$$

where $V$ = median volume of surface adenomata in mm³ and $t$ = time in weeks PU. This equation can be simplified to:

$$V = 0{\cdot}000776 \times t^{2{\cdot}181}$$

The specific growth rate ($k_G$) of the adenomata, defined as the growth rate per unit volume,

$$\frac{1}{V}\cdot\frac{dV}{dt},$$

is given by differentiating log $V$ with respect to $t$:

$$k_G = \frac{d(\log V)}{dt}$$

$$= \frac{1}{V}\cdot\frac{dV}{dt}$$

$$= \frac{2{\cdot}181 \times 0{\cdot}000776\, t^{1{\cdot}181}}{0{\cdot}000776\, t^{2{\cdot}181}}$$

*i.e.*

$$k_G = \frac{2{\cdot}181}{t}.$$

This equation shows that the growth rate of the adenomata decreases with time.

*Adenoma metaphase and labelling indices*

Figure 4 shows that both the native metaphase index ($I_M$) and the 4 h Col-

FIG. 4.—Metaphase index of adenomata in relation to survival.

FIG. 5.—Labelling index of adenomata in relation to survival.

cemid metaphase index ($I_{M(a)}$) decrease with increasing age of the adenomata. Since $I_{M(a)}$ is an index of the rate of entry into metaphase ($R_M$), the birth rate ($k_B$) or rate of adenomata cell production is also decreasing. Similarly, $I_S$ and $I_{DL}$ fall with time (Fig. 5). The difference between the two curves ($I_{DL}$ — $I_S$) likewise decreases, indicating that $R_S$ falls with age. The Table gives the values of $R_M$, $R_S$, $I_M$ and $I_S$ as well as the calculated values of $t_M$ and $t_S$.

*Female mice*

There were no significant differences between adenomata occurring in males and females at 26 weeks PU. The mean number of surface adenomata per left lung in urethane treated mice was 15 for males and 14 for females. The median sizes of the surface adenomata in both sexes were similar. The proliferative rates of the adenomata as shown by the $R_S$ and $R_M$ values were slightly higher in females than in males, but these differences were not significant. The only difference between the sexes was in their weights at death, the average

FIG. 6.—Metaphase index of alveolar tissue in relation to survival: (a) arrested metaphases; (b) native metaphases.

FIG. 7.—Labelling index of alveolar tissue in relation to survival: (a) single indices; (b) double indices.

weight of females being 34 g compared with 41 g for males, a difference probably due to the larger fat deposits in males. These observations support the conclusions of Shimkin (1955) that urethane induced adenomata are not influenced by the sex hormonal environment. All the cell kinetic data from males and females were accordingly combined for the 26-week interval.

*Alveolar tissue*

Figure 6 shows no significant differences between $I_{M(a)}$ in experimental and control mice. However, there is a urethane effect on $I_M$ since the experimental values are all higher than those of the corresponding controls. The experimental $I_M$ values also show a peak response at 2-weeks PU whereas the control values remain approximately constant with time.

The labelling indices (Fig. 7) reveal increased proliferative activity in the first month after urethane treatment. Both $I_S$ and $I_{DL}$ in the experimental mice have peak values at 2 weeks PU but return to control levels by 2 months PU. However, the labelling indices exhibit an anomaly, in that $I_S$ obtained from Colcemid treated mice is on the whole higher than $I_{DL}$. This phenomenon is evident in control mice at 0, 2, 4 and 9 weeks and in experimental ones at 4, 17 and 26 weeks PU. Only in the experimental peak values at 1, 2 and 3 weeks PU is $I_{DL}$ larger than $I_S$, as might be expected throughout. At the 26-week interval no differences were evident between males and females for any of the alveolar tissue parameters, and the values were therefore combined into a single group.

DISCUSSION

*Adenomata*

The urethane induced surface adenomata increase in size in such a way that their growth from 4 to 32·5 weeks PU conforms, as already indicated, to a quadratic equation: $V = 0{\cdot}000776 \times t^{2{\cdot}181}$. However, with such a simple growth equation extrapolation beyond the limits of 4 and 32·5 weeks is not justified. Surface adenomata display a decrease in growth rate as they become older. The change in growth rate could be due to a decrease in $k_B$ and/or an increase in $k_L$ but no areas of necrosis were apparent throughout the whole observation period. The decreases in $R_M$

FIG. 8.—Alveolar tissue nuclear count in relation to survival.

TABLE.—*Kinetic Parameters of Adenomata in Relation to Survival*

| Time PU (weeks) | *$R_M$ % per h | $I_M$ % | $t_M$ h | $R_S$ % per h | $I_S$ % | $t_S$ h |
|---|---|---|---|---|---|---|
| 4 | — | 0·88 | — | 3·0 | 2·8 | 0·93 |
| 9 | 0·185 | 0·15 | 1·35 | 1·2 | 1·8 | 1·5 |
| 17 | 0·075 | 0·24 | 0·32 | 0·8 | 1·4 | 1·75 |
| 26 | 0·055 | 0·09 | 1·64 | 0·3 | 1·2 | 4·0 |

* Calculated on the assumption that Colcemid does not arrest cells already in metaphase at the time of its administration.

and $R_S$ demonstrate that $k_B$ is falling with time, which could in turn be caused by a decrease in growth fraction ($I_P$) and/or an increase in cell cycle time ($t_C$). The Table shows that $t_S$ increased markedly with time, suggesting that $t_C$ could also be increasing. However, the value of 1–4 h for $t_S$ is short in comparison with that of 5–10 h given by Cleaver (1967) for mouse tissues. From the Table it can be seen that $R_S$ differs from $R_M$ by factors of between 5 and 11. It is possible that diurnal effects are contributing towards this discrepancy. However, Bertalanffy *et al.* (1965) could find no evidence from their work, or from the literature, for any diurnal variation in proliferative activity in experimental animal tumours. It is also pertinent that Bertalanffy (1964) found no diurnal variation in mitotic rate in normal rodent alveolar wall cells. Another possibility is that Colcemid is somehow interfering with labelling. This is unlikely since Puck and Steffen (1963) showed that Colcemid had no effect on the G1, G2 and S phases of HeLa cells *in vitro*. The discrepancy between $R_S$ and $R_M$ is the subject of further investigation.

Surface adenomata could represent a faster growing sub-population appearing early on the lung surface, and they might not be comparable with sectioned adenomata, from which metaphase and labelling indices were obtained. However, this objection is unlikely for several reasons. Stewart (1959) noted that most adenomata are subpleural in origin and subsequent growth makes them readily apparent on the lung surface. Our use of a dissecting microscope and strong reflected light permitted recognition of adenomata when they were no larger than 150–200 $\mu$m in diameter and situated at some depth in the lung parenchyma. Moreover, Shimkin and Polissar (1955) estimated from serial lung sections that, by 105 days PU, 50% of the adenomata in mouse lungs were visible on the lung surface to the naked eye alone. The growth pattern exhibited by our surface adenomata correlates well with that observed by Shimkin and Polissar (1955) for serially sectioned adenomata. All these considerations suggest that the surface adenomata do in fact accurately reflect the growth of the whole adenoma population of the lung.

### *Alveolar wall tissue*

An increase in proliferative rate occurs within the first month following urethane treatment. This response reaches a peak at 2 weeks but disappears by about 2 months PU. During this response $I_{DL}$, $I_S$ and $I_M$ are all elevated but no corresponding increase in $I_{M(a)}$ could be detected. Shimkin and Polissar (1955) noted a progressive increase in the number of alveolar wall cells 1–28 days after urethane injection; a diffuse hyperplasia and small subpleural clusters of enlarged cells were found after 21 days but the latter gradually disappeared as adenomata became apparent. Foley *et al.* (1963) demonstrated an increased DNA synthetic index in alveolar tissue 2–7 days following urethane injection; this proliferative response and the numbers of adenomata induced were decreased by x-irradiation of the lungs before urethane treatment. These observations raise the question as to whether the urethane induced proliferative response is a necessary precursor of adenomata as the results of Foley *et al.* (1963) suggest. The appropriate cell population is not, however, being studied since adenomata are derived from type II alveolar epithelial cells (Svoboda, 1962), whereas alveolar walls also include type I cells, interstitial cells and endothelium. The majority of cells taking part in the urethane induced proliferative response in alveolar walls appear to be of the non-vacuolated type, possibly replacing cells killed by the toxic effects of urethane (Kaufmann, 1969). Shimkin *et al.* (1969) believed that the proliferative response in alveolar walls was a side-effect unrelated to urethane carcinogenesis, since urethane doses of 0·25 mg and 1·0 mg/g body weight induced adenomata but only the latter dose increased the pulse labelling index in alveolar walls. However, Kauffman (1974) has recently shown that there is a doubling in the size of the type I, type II and alveolar macrophage populations from 2 to 6 weeks after the beginning of chronic exposure to urethane. Thus, growth of type II cells is evidently a component of the hyperplasia accompanying urethane exposure.

$I_S$ was often elevated above $I_{DL}$ in alveolar walls (Fig. 7), making it impossible to calculate $R_S$ and $t_S$, but in the adenomata $I_{DL}$ was consistently above $I_S$ (Fig. 5). It is therefore unlikely that Colcemid is interfering with DNA synthesis and hence with labelling. When the nuclear count of alveolar tissue is plotted against time (Fig. 8) Colcemid is seen to cause an increase in cellularity. There was no suggestion that Colcemid caused collapse of lung and so augmented the cell population per field. On the other hand, Colcemid might promote immigration of circulating cells from the blood into the alveolar wall. Dixon and Malden (1908) reported that colchicine produced a leucocytosis in rabbits and dogs, while vincristine caused a rapid leucocytosis and a decrease in the cellularity of the bone marrow in rats (Frei *et al.*, 1964). Stathmokinetic agents could thus cause a rapid release of leucocytes from the bone marrow into the blood and some leucocytes might be sequestered in the alveolar tissue. When $^3$H-TdR is given at the same time as Colcemid, some of these leucocytes might become labelled if they were immature and still synthesizing DNA. The increase in the single labelling index of alveolar wall tissue (Fig. 7) may be explained in this way.

This work was supported by a grant awarded to A. G. H. by the North of England Council of the Cancer Research Campaign. We are indebted to Dr David Appleton for mathematical advice.

### REFERENCES

BERTALANFFY, F. D. (1964) Respiratory Tissue: Structure, Histophysiology, Cytodynamics. II. New Approaches and Interpretations. *Int. rev. Cytol.*, **17**, 213.

BERTALANFFY, F. D., SCHACHTER, R., ALI, J. & INGIMUNDSON, J. C. (1965) Mitotic Rate and Doubling Time of Intraperitoneal and Subcutaneous Ehrlich Ascites Tumor. *Cancer Res.*, **25**, 685.

CLEAVER, J. E. (1967) *Thymidine Metabolism and Cell Kinetics*. Ed. A. Neuberger and E. L. Tatum. Amsterdam: North-Holland.

DIXON, W. E. & MALDEN, W. (1908) Colchicine with Special Reference to its Mode of Action and Effect on Bone Marrow. *J. Physiol.*, **37**, 50.

FOLEY, W. A., COLE, L. J., INGRAM, B. J. & CROCKER, T. T. (1963) X-ray Inhibition of Urethan-stimulated Proliferation of Lung Cells of Mouse as Estimated by Incorporation of Tritiated Thymidine. *Nature, Lond.*, **199**, 1267.

FREI, E., WHANG, J., SCOGGINS, R. B., VAN SCOTT, E. J., RALL, D. P. & BEN, M. (1964) The Stathmokinetic Effect of Vincristine. *Cancer Res.*, **24**, 1918.

KAUFFMAN, S. L. (1969) Alterations in Cell Proliferation in Mouse Lung following Urethane Exposure. 1. The Nonvacuolated Alveolar Cell. *Am. J. Path.*, **54**, 83.

KAUFFMAN, S. L. (1974) Kinetics of Alveolar Epithelial Hyperplasia in Lungs of Mice Exposed to Urethane. 1. Quantitative Analysis of Cell Populations. *Lab. Invest.*, **30**, 170.

NETTLESHIP, A., HENSHAW, P. S. & MEYER, M. L. (1943) Induction of Pulmonary Tumors in Mice with Ethyl Carbamate (Urethane). *J. natn. Cancer Inst.*, **4**, 309.

PUCK, T. P. & STEFFEN, J. (1963) Life Cycle Analysis of Mammalian Cells. I. A Method for Localising Metabolic Events within the Life Cycle, and its Application to the Action of Colcemide and Sublethal Doses of X-irradiation. *Biophys. J.*, **3**, 379.

ROGERS, S. (1951) Age of the Host and Other Factors Affecting the Production with Urethane of Pulmonary Adenomas in Mice. *J. exp. Med.*, **93**, 427.

SHIMKIN, M. B. (1955) Pulmonary Tumors in Experimental Animals. *Adv. Cancer Res.*, **3**, 233.

SHIMKIN, M. B. & POLISSAR, M. J. (1955) Some Quantitative Observations on the Induction and Growth of Primary Pulmonary Tumors in Strain A Mice receiving Urethan. *J. natn. Cancer Inst.*, **16**, 75.

SHIMKIN, M. B., SASAKI, T., McDONOUGH, M., BASERGA, R., THATCHER, D. & WEIDER, R. (1969) Relation of Thymidine Index to Pulmonary Tumor Response in Mice receiving Urethane and Other Carcinogens. *Cancer Res.*, **29**, 994.

STEWART, H. L. (1959) Pulmonary Tumors in Mice. In *The Physiopathology of Cancer*. 2nd Edn Ed. F. Hamburger. New York: Cassell.

SVOBODA, D. J. (1962) Ultrastructure of Pulmonary Adenomas in Mice. *Cancer Res.*, **22**, 1197.